# On the Treatment of Bilious Remitting Fever

**Published:** 1846-02

**Authors:** Abraham Livezey

**Affiliations:** Lumberville, Bucks County, Pennsylvania


*On the Treatment of Bilious Remitting Fever.* By ABRAHAM LIVEZEY, A. M., M. D., of Lumberville, Bucks County, Pennsylvania. Communicated in a letter to the Editor.

During the past summer a bilious remittent fever raged in many parts of New Jersey, especially in Hunterdon county, about Ringoes, Head Quarters, Mount Airy, &c., where it proved alarmingly fatal—30 dying of the first 80 cases. These were treated after the old method of managing febrile diseases, by a "mixed, sedative and perturbating treatment." Purgatives, frequently given; small doses of calomel, variously combined, throughout the disease; cold water prohibited under false apprehensions; general depletion resorted to, to the entire exclusion of cups. This is the synopsis of the mode of treatment practised at the forementioned places, as obtained from one of the physicians (ætat. 80) residing in the vicinity of those places, and who met me in my first case, after the epidemic had extended within the limits of my practice.

This case, a young, robust farmer, had the full benefit of the old, or rather of Prof. Eberle's practice, viz.: cal., antim., ipecac. and nitre, variously combined, and in small doses, till the case terminated fatally at the close of the third week.

After his death, several of the same family, and many in the vicinity afflicted in like manner, were solely entrusted to my care, and all successfully treated by bearing in mind the pathology of the gastro-enteric mucous membrane, and pursuing the "milder, more soothing, and more philosophical treatment," as recommended by Prof. Dunglison in his Practice of Medicine, and inculcated in his lectures at the Alms-house.

High temperature and vascular action were allayed by sponging the surface with vinegar and water, and cold water freely allowed internally; the irritated and inflamed gastro-enteric mucous membrane was soothed by mucilaginous drinks; hyperæmia of any organ was promptly met by cups, after which, if tenderness of the bowels upon pressure persisted, blisters were applied. The bowels were daily unloaded by means of a small quantity of ol. ricini, aided by soothing or stimulating enemata, as the case required. And by these means I have had the pleasure of restoring to perfect health 19 cases in New Jersey, and 12 on this side of the Delaware, being all the cases that I have had under my care.

